# Reply to Srivastava and Gumbo, “Rigor and reproducibility of hollow-fiber infection model for antiviral pharmacokinetics/pharmacodynamics studies”

**DOI:** 10.1128/aac.00980-26

**Published:** 2026-09-03

**Authors:** Lalitya M. Sudarsono, Suzanne A. M. Wenker, Xuanlin Liu, Jorn Brink, Dirk-Jan van den Berg, J. G. C. van Hasselt, Anne-Grete Märtson

**Affiliations:** 1 Division of Systems Pharmacology and Pharmacy, Leiden Academic Centre for Drug Research, Leiden University 4496, Leiden, the Netherlands; Providence Portland Medical Center, Portland, Oregon, USA

## REPLY

We thank Srivastava and Gumbo for their interest (1) in our paper describing a hollow fiber infection model (HFIM) for cytomegalovirus (CMV) pharmacokinetic/pharmacodynamic (PK/PD) studies (2). Their comments allow us to clarify several aspects of the setup.

The requirement stated by Srivastava and Gumbo of 43 L of cell culture medium and 4.3 L of fetal bovine serum (FBS) per system per day is based on an incorrect assumption. The pump was assumed to have an inflow rate of 30 mL/min. However, this rate actually refers to the HFIM circulation loop, which maintains rapid equilibrium between the central compartment and the cartridge. This does not consume any medium. Medium consumption is set by the clearance rate, which, as described in the Materials and Methods section, corresponds to approximately 577 µL/min. This is equivalent to 0.83 L of medium, and 83 mL of FBS per day. We agree that the cartridge is not the only cost, but it is the principal single-use component, and the use of a hemodialyzer saves substantially over commercial cartridges, as each experimental arm needs its own cartridge.

Regarding cell viability, the sustained increase in CMV DNA copies and infectious titer over the 6-day infection period indicates that the cell monolayer remained capable of supporting active viral replication throughout. Concerning drug-related cytotoxicity, preliminary testing of ganciclovir in a static plate system over 14 days without medium replacement showed full MRC-5 viability above 10 mg/L, which indicates that exposures achieved in the HFIM remained below the cytotoxicity threshold.

Considering comments on the concentration-time profiles in our HFIM study, sampling was performed every 24 h on three separate days rather than every 12 h, as a 12-h schedule would have required overnight sampling. This accounts for the absence of measured points for the second dose in Figure 3. The intended *C*_max_ of 10 mg/L was not fully reached. Such deviations between intended and achieved profiles are expected in an experimental model, which is why we measured ganciclovir concentrations in real time rather than relying on a simulated PK profile. The observed elimination rate constant of 0.11 h⁻¹ corresponds to a half-life of approximately 6.3 h, which is indeed higher than the theoretical target value. We agree that reporting this explicitly in Table 1 would have been clearer. Drug clearance was nonetheless estimated with good precision (0.033 L/h, RSE 0.2%), and the resulting AUC_0−12h_ of 42.2 mg·h/L falls within the clinically relevant target range (3). The 8-h window in Figure 4B was selected to demonstrate equilibrium between the intracapillary and extracapillary spaces and includes the trough concentration; longer PK profiles can be generated as required.

We thank the authors for identifying the labeling error in Figure 4D. The value shown is the AUC calculated from the 7-h profile in Figure 4B, not a 24-h AUC. This concerns the label only and does not affect the AUC_0−12h_ estimate in Table 1.

We agree that inter-unit variability estimates, e.g., in viral titers, are important. The present study used a limited number of replicates per condition, as we view our study as an initial proof-of-principle study establishing a CMV HFIM model rather than a definitive PK/PD analysis. Similarly, we fully agree and support the importance of clear quality control and reporting standards for the HFIM, which are well established for antibacterial applications (4). Comparable standards for antiviral work remain far less developed, but we hope our publication contributes to this goal.

In conclusion, we remain confident in the feasibility of a hemodialyzer-based cartridge as an alternative to commercial systems that is capable of supporting CMV infection and reproducing a clinically relevant ganciclovir exposure profile. Given the urgent need for antiviral PK/PD studies, a low-cost hollow-fiber setup can improve accessibility for academic research.
